# Intravenous Amantadine for Freezing of Gait Resistant to Dopaminergic Therapy: A Randomized, Double-Blind, Placebo-Controlled, Cross-Over Clinical Trial

**DOI:** 10.1371/journal.pone.0048890

**Published:** 2012-11-19

**Authors:** Young Eun Kim, Ji Young Yun, Hui June Yang, Han-Joon Kim, Namyi Gu, Seo Hyun Yoon, Joo-Youn Cho, Beom S. Jeon

**Affiliations:** 1 Department of Neurology and Movement Disorder Center, Seoul National University Hospital, Seoul, Korea; 2 Department of Clinical Pharmacology and Therapeutics, Seoul National University College of Medicine and Hospital, Seoul, Korea; INSERM/CNRS, France

## Abstract

**Background:**

Freezing of gait (FOG) is one of the most disabling symptoms in Parkinsonism. Open-label studies have suggested that intravenous (IV) amantadine is effective against FOG resistant to dopaminergic therapy in Parkinson's disease (PD). We evaluated the efficacy of IV amantadine on FOG resistant to dopaminergic therapy.

**Methodology/Principal Findings:**

This was a randomized, double-blind, placebo-controlled, cross-over study on IV amantadine. The placebo (normal saline) and amantadine (400 mg/day) were injected for 2 days with a 52-hour washout period. The instruments for the outcome measures were the Freezing of Gait Questionnaire (FOGQ), Unified Parkinson's disease rating Scale (UPDRS), and the duration of the 4×10 m walking test. The placebo arm was compared to the amantadine arm. Ten patients were enrolled but two patients withdrew, one from each arm. The FOGQ and UPDRS scores and the duration of the 4×10 m walking test improved in both arms compared to the baseline (*P*<0.05 in all). However, there were no differences in these values between the amantadine arm and placebo arm (*P* = 0.368, *P* = 0.583, *P* = 0.206, respectively). Follow-up measures 2weeks after discharge in an open-label study showed the beneficial effects of an amantadine tablet on FOG (FOGQ, *P* = 0.018; UPDRS, *P* = 0.012 respectively).

**Conclusions/Significance:**

This double blind, placebo-controlled study did not show the efficacy of IV amantadine on FOG when compared with the placebo. This study provides Class II evidence due to small sample size for the lack of benefit of IV amantadine on FOG resistant to dopaminergic therapy

**Trial Registration:**

Clinicaltrials.gov NCT01313819

## Introduction

Freezing of gait (FOG) is one of the most disabling symptoms in Parkinsonism [1]. Nearly one-third of Parkinson's disease (PD) patients experience some type of freezing episode [2]. FOG interferes with daily activities, increases the risk of falling over, and contributes significantly to an impaired quality of life (QOL) [3], [4]. Even though the mechanism of FOG is understood in part, treatment is often ineffective, especially in dopaminergic drug resistant FOG except some methods using rehabilitation and cues [5]–[8].

Amantadine has been used for the treatment of PD since the late sixties [9], [10], and the rapid effect of intravenously applied amantadine on PD motor signs has been acknowledged [11]. Recently it has received attention for the treatment of levodopa-induced dyskinesia [12]. There are only few studies for the effectiveness of amantadine on FOG [10] One retrospective study reported that patients who were treated with amantadine were less likely to develop FOG [13]. Another study reported that amantadine decreased FOG in patients with progressive supranuclear palsy (PSP) and pure akinesia (PA) [14]. However, one retrospective analysis showed that the combination treatment of L-dopa and amantadine had a higher frequency of FOG [15]. Our preliminary study with intravenous (IV) amantadine showed that it might be effective in dopaminergic resistant FOG mainly in PD [16]. However, this study had an open and uncontrolled design that made it susceptible to the placebo effect. Therefore, we did a randomized, double-blind, placebo-controlled, cross-over study to examine the effect of IV amantadine on drug resistant FOG.

## Methods

The protocol for this trial and supporting CONSORT checklist are available as supporting information; see Checklist S1 and Protocol S1.

### 1. Patients and design

We recruited patients ranging in age from 30 to 80 years who were diagnosed with Parkinson's disease using the UK Parkinson Disease Brain Bank Criteria and with intractable FOG between April 2011 and May 2011 at the Movement Disorder Center at Seoul National University Hospital (SNUH). Intractable FOG was defined as a FOG questionnaire (FOGQ) score of ≥10 [16] and that FOG persisted during the ‘On’ period even when high doses of dopaminergic medication were given. All medications were kept stable for 1 month before the start of the study. The following exclusion criteria were applied: 1) Parkinson plus syndrome and secondary Parkinsonism; 2) patients who received amantadine within 1 month; 3) dementia or psychiatric problems; 4) severe medical disease, especially chronic renal failure.

**Figure 1:**
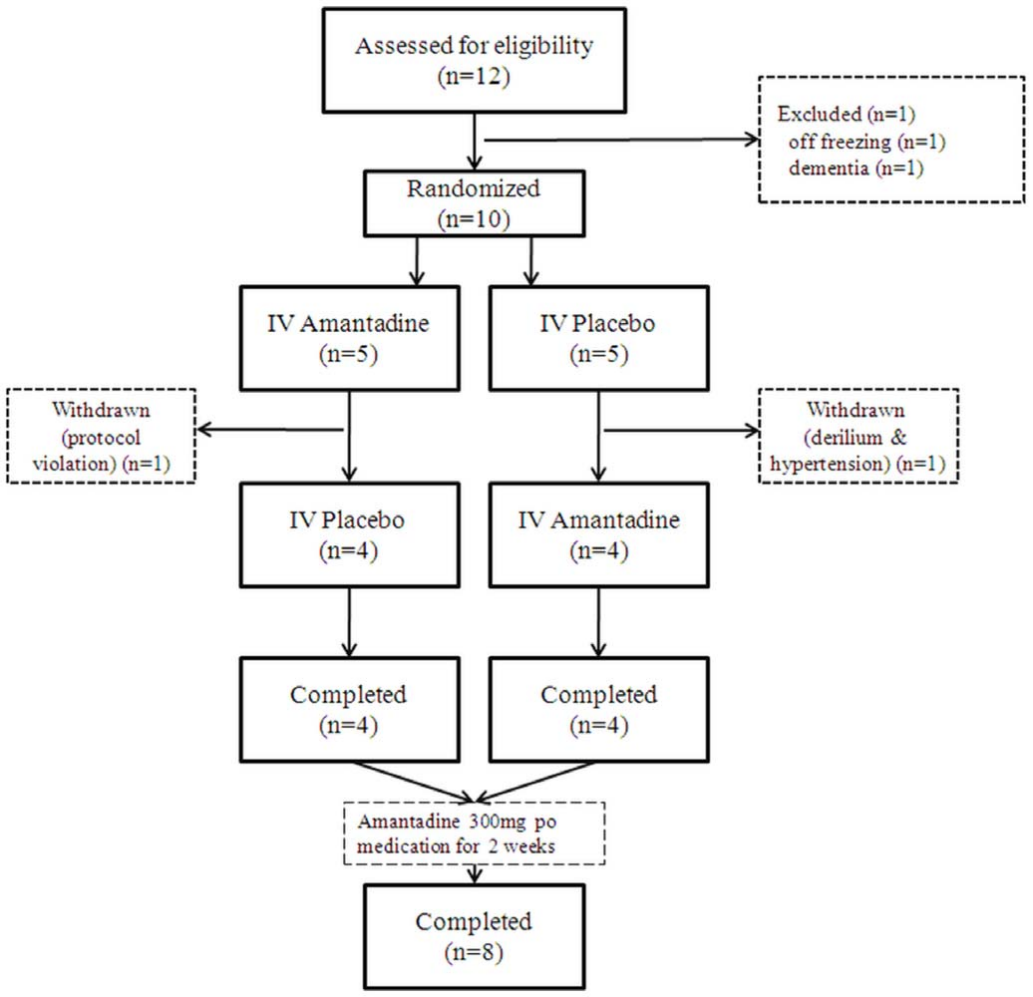
CONSORT diagram.

**Figure 2:**
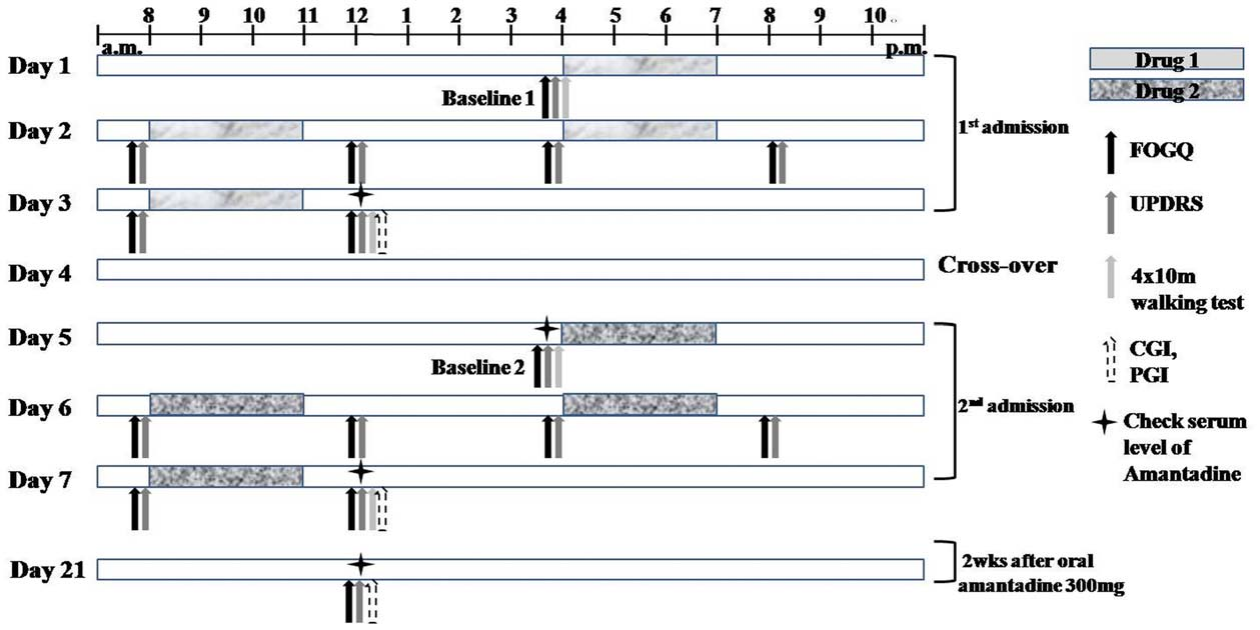
Timeline of drug and clinical assessments.

The study followed a double blind, placebo-controlled, cross-over design (Figure 1). All patients were admitted to the Clinical Trial Center at Seoul National University Hospital (SNUH). Randomization was done by the Medical Research Collaborating Center (MRCC) at SNUH. The randomization table made by the MRCC was transferred directly to the pharmacy at the Clinical Trial Center. All subjects, caregivers, and investigators except for pharmacists were blinded from assignment until all study has been completed. All subjects were admitted twice for 3 days with a 52-hour washout period between each admission. IV amantadine or placebo was assigned to each admission by random order according to the randomization table. Blood pressure, ECG, and renal function were monitored. All patients were prescribed amantadine 100 mg tablet t.i.d. at discharge in an open-label fashion, and were followed up after 2 weeks (Figure 2).

All studies were carried out with the subjects having an adequate understanding and having given their informed consent. This study protocol was approved by the institutional review board at our institution. The clinical trial identifier number assigned by clinicltrial.gov was NCT01313819.

### 2. Dosage schedule

Our standard regimen was amantadine at 200 mg in 500 ml of normal saline solution or 500 ml of normal saline as the placebo along with the pre-existing dopaminergic and non-dopaminergic medication. The bags containing amantadine and placebo were identical. IV drugs were infused in the subjects 4 times for 3 hours at 4 p.m. of the 1^st^ day, at 8 a.m. and 4 p.m. of the 2^nd^ day, and at 8 a.m. of the 3^rd^ day. All patients took their first oral medications at 7 a.m. before breakfast.

### 3. Clinical assessment

All subjects underwent a baseline assessment using the FOGQ [17] and Unified Parkinson's Disease Rating Scale (UPDRS) scores, the 4×10 m walking test (measuring the time while walking a 10 m length passage, 4 times), a Mini Mental Status Examination (MMSE), and a Frontal Lobe Assessment Battery (FAB). The FOGQ and UPDRS motor scores were assessed at 8 a.m., 12 p.m., 4 p.m., and 8 p.m. of the 2^nd^ day and at 8 a.m. and 12 p.m. of the 3^rd^ day for each admission. FOGQ was based on the status of FOG over the previous 7 days at baseline 1, and over the 4 hours prior to each scoring during the admission. Clinical improvement of the Clinical Global Impression scale (CGI), Patient Global Impression scale (PGI) and 4×10 m walking test were checked before discharge [18] The serum level of amantadine [19] was checked at 12 p.m. before discharge from the 1^st^ and 2^nd^ admission and at 4 pm before injection of the drug at the 2^nd^ admission. The FOGQ and UPDRS scores, CGI, PGI, and amantadine serum levels were checked at the 3^rd^ visit on Day 21 (Figure 2).

### 4. Statistical assessment

Based on the results of our previous study, [16], the target sample of eight patients was expected to detect a reduction in the FOGQ score by at least 30% with a 1-sided p value (α) of .05 and 80% power. In the previous study, PD patients who responded showed a mean improvement of 30% on the FOGQ score after receiving IV amantadine.

The relative change in 6 repeated measured data of the FOGQ and UPDRS 14, 15, III scores were analyzed using the repeated measures ANOVA (between the amantadine arm and placebo arms, between 1^st^ admission and 2^nd^ admission, and placebo arm value according to the order of amantadine and placebo). We used Friedman test for more than 3 groups for the comparison of the baseline or last values of the FOGQ and UPDRS scores and the mean value of the FOGQ and UPDRS scores that were measured during each admission. And we used Wilcoxon signed rank test for single measured data of 2 groups (last UPDRS 14, 15 score and amantadine serum level between amantadine arm and placebo arm; UPDRS III and FOGQ between baseline and 2 weeks after study completion).

We used Mann-Whitney *U*-test for the comparison of 4×10 m walking test value in placebo arm according to order of amantadine and placebo. These statistical analyses were conducted using software IBM SPSS statistics, ver. 19.0.

## Results

A total of 10 patients were recruited and randomized. Eight subjects completed the entire study (Figure 1). One patient withdrew from each arm of the 1^st^ admission for the following reasons: one patient in the amantadine arm was withdrawn due to a protocol violation because one patient took amantadine tablets secretly; one patient in the placebo arm was withdrawn due to delirium and hypertension, which continued for 3 hours on the 1^st^ night of the 1^st^ admission and fully recovered without any further complications. In the end, 4 male and 4 female subjects completed the study. All other baseline clinical features are summarized in Table S1.

The FOGQ score, UPDRS part II items 14 (freezing) and 15 (walking) score, UPDRS part III score, and the duration of the 4×10 m walking test were compared between the baseline and the amantadine and placebo arms. Compared to the baseline values, the mean FOGQ and UPDRS III scores and the last UPDRS score and FOGQ score were significantly improved in the amantadine arm and the placebo arm. On the other hand, there were no differences in the FOGQ and UPDRS scores between the amantadine and placebo arms and the duration of the 4×10 m walking test was not differ between baseline, amantadine, and placebo arm (Table 1).

**Table 1:** The effect of treatment (n = 8).

				*P* value	
Variables	B (n = 8)	A (n = 8)	P (n = 8)	B vs. A vs. P	A vs. P
Mean UPDRS III	23.3±7.3*	19.5±6.4	19.3±6.6	.03†	.583§, .654∥
Mean UPDRS 14		1.9±0.7	1.7±0.4	.235‡	.213§, .091∥
Mean UPDRS 15		1.7±0.6	1.5±0.5	.310‡	.487§, .606∥
Mean FOGQ	16.9±3.6*	10.8±3.2	9.1±2.6	.005†	.368§, .231∥
4×10 *m* walking test (sec)	81.8±58.9	56.6±17.7	99.8±88.8	.206‡	
Last UPDRS III	23.3±7.3*	19.4±7.7	18.2±7.0	.004†	
Last UPDRS 14		1.6±0.7	1.8±0.7	.655‡	
Last UPDRS 15		1.6±0.9	1.5±0.8	.655‡	
Last FOGQ	16.9±3.6*	10.6±4.0	9.4±4.2	.004†	
Amantadine serum level (ng/ml)		1169.1±262.6 (897.5–1587.0)	13.6±14.4 (0–41.5)	.012‡	

Abbreviations: B = Baseline; A = Amantadine arm; P = Placebo arm;

UPDRS = Unified Parkinson's Disease Rating Scale score; UPDRS 14 = UPDRS item 14 freezing; UPDRS 15 = UPDRS item 15 gait; FOGQ = Freezing of gait questionnaire score.

B (baseline) data and 10×4 m walking test are single measured results.

Data of A and P are 6 repeated data before and 1 hr after injection (refer to figure 2).

* P<.05, B vs. A and B vs. P.

† Friedman test.

‡ Wilcoxon signed rank sum test for A vs. P.

§ Repeated measures ANOVA for total 12 repeated measures of A and P; 6 repeated measures for each arm.

∥ Repeated measures ANOVA only for data at 1 hrs after injection (total 6 repeated measures of A and P, 3 repeated measures for each arm).

We compared the results of the baseline with the 1^st^ and 2^nd^ admissions irrespective of the treatments to determine the effect of the admission itself and the order effect. Compared to the baseline values, the mean and the last scores for FOGQ and UPDRS were improved in both the 1^st^ and 2^nd^ admissions with statistical significance. There was no significant difference in the FOGQ and UPDRS scores between the 1^st^ and 2^nd^ admissions (Table 2).

**Table 2:** Effect of order (comparison of baseline vs. 1^st^ and 2^nd^ admission results) (n = 8).

					*P* value	
Variables	B1	Ad1	B2	Ad2	B1 vs.Ad1 vs. (B2) vs.Ad2	Ad1 vs. Ad2
Mean UPDRS III	23.3±7.3*	19.0±6.6	19.9±8.1	19.8±6.4	.019†	.256‡, .415§
Mean FOGQ	16.9±3.6*	9.4±3.2		10.5±2.8	.006†	.415‡, .513§
Last UPDRS III	23.3±7.3*	18.1±7.9	19.9±8.1	19.5±6.8	.012∥	
Last FOGQ	16.9±3.6*	9.0±4.3		11.0±3.6	<.001∥	

Abbreviations: B1 = Baseline 1; Ad1 = 1^st^ admission; B2 = baseline 2; Ad2 = 2^nd^ admission; Other abbreviations are same as in Table 1.

B1 and B2 are single measured data.

Last UPDRS III and Last FOGQ are single measured data.

* P<.05 when comparing with Ad1, (B2), Ad2 respectively using Wilcoxon signed rank sum test.

† Friedman test.

‡ Repeated measures ANOVA for total 12 repeated measure of Ad1 and Ad2 (6 repeated measures for each arm).

§ Repeated measures ANOVA only for data at 1 hr after injection (total 6 repeated measures of Ad1 and Ad2, 3 repeated measures for each arm).

∥ Repeated measures ANOVA for B1, B2, and last values of Ad1 and Ad2.

We compared the values of the placebo arms in the two groups; AP group (Amantadine on 1^st^ admission and then Placebo on 2^nd^ admission) and PA group (Placebo on 1^st^ admission and then Amantadine on 2^nd^ admission) since the residual effect of amantadine might affect the results of the 2^nd^ admission in the AP group. Our hypothesis was that the FOGQ or UPDRS scores would be better in the placebo arm (2^nd^ admission) of the AP group if the effect of amantadine persisted during the 2^nd^ admission. However, the UPDRS and FOGQ scores of the placebo arms did not differ between the AP and PA groups, and the duration of the walking test rather tended to be longer in the AP group than in the PA group (Table 3).

**Table 3:** Comparison of placebo arm values according to order of amantadine and placebo.

Values in Placebo arm	AP group (n = 4)	PA group (n = 4)	*P* value
Mean UPDRS III	22.5±6.5	16.2±5.7	.194*, .173†
Mean FOGQ	10.4±3.3	7.8±1.0	.168*, .169†
4*10 *m* Walking test (sec)	148.0±110.3	51.5±5.4	.057‡
Amantadine serum level before IV injection in 2^nd^ admission (ng/ml)	126.3±70.9 (73.6–229.0)	0	

Abbreviations: AP group = Amantadine on 1^st^ admission and then Placebo on 2^nd^ admission; PA group = Placebo on 1^st^ admission and then Amantadine on 2^nd^ admission; Other abbreviations are same as in Table 1.

* Repeated measures ANOVA for total 6 repeated data of placebo arm of each group.

† Repeated measures ANOVA only for data at 1 hr after injection (total 3 repeated data of placebo arm of each group).

‡ Mann-Whitney *U* test.

The PGI questionnaire reported that placebo was better in 2 patients; the effect was similar in both the placebo and amantadine in 3 patients, and amantadine was better in 3 patients. In the CGI questionnaire, the placebo was better in 3 patients; the effect was similar in both the placebo and amantadine in 1 patient, and amantadine was better in 4 patients.

After the study, all subjects were given 100 mg amantadine tablets t.i.d. for 2 weeks. FOGQ and UPDRS scores improved significantly in all patients compared to the baseline score (n = 8, *P* = 0.018 and 0.012 respectively, amantadine serum level = 920.0±377.7 ng/ml).

### Side effect profiles

In the amantadine arm, 1 patient had hypertension and 1 patient had hypotension. In the placebo arm, 1 patient had delirium and hypertension who was withdrawn and 1 patient had hypertension. All subjects made a full recovery without residual complications. There was no worsening in renal function.

## Discussion

The purpose of this study was to evaluate the effect of IV amantadine on dopaminergic resistant FOG. Our previous open-label study suggested the benefits of IV amantadine on FOG in patients with Parkinson's disease [16]. Contrary to the previous study, this double blind, placebo-controlled study did not show the efficacy of IV amantadine compared to placebo.

Although the serum amantadine level was in the therapeutic range in the amantadine arm [19], the FOGQ and UPDRS scores and the duration of the 4×10 m walking test did not differ between the two arms. However, compared with the baseline results, all variables including the FOGQ and UPDRS scores and the duration of the 4×10 m walking test were improved in the 1^st^ admission and 2^nd^ admission regardless of the amantadine and placebo arms. This may suggest the effect of the admission itself. One of the unique features of FOG is its considerable variability [17], which makes it difficult to evaluate the severity of FOG objectively. Alternatively, it may suggest that the placebo effect of the IV drug is larger than the therapeutic effect within 2 days of administrating the drug, which makes it difficult to prove the effect of the drug.

Among the 8 patients, four subjects had peak dose dyskinesia and 1 subject had peak and diphasic dyskinesia. Three out of the 5 patients with dyskinesias showed a decrease in dyskinesia during the amantadine but not placebo infusion; however, the FOGQ score was worse during the amantadine infusion in these three patients. This means that the antidyskinetic mechanism of amantadine, known as the NMDA receptor antagonist, did not improve the freezing of gait [20].

However, when amantadine 300 mg was prescribed for 2 weeks in an open-label fashion, oral amantadine was effective for FOG based on FOGQ and UPDRS part III. Moreover, 7 out of 8 patients reported improvement in the FOG on questionnaire for PGI. A previous crossover study using amantadine in patients with Parkinson plus showed the benefits for FOG after 4 weeks of taking amantadine tablets (150 mg) [14]. Although it is known that IV amantadine induces a rapid improvement in parkinsonian motor symptoms, a delay in the motor benefits may explain the inadequate benefits of short term IV amantadine and the benefit of oral amantadine for 2 weeks on FOG [11].

Amantadine usually reached peak plasma levels between 1 and 7 hours following a single oral dose [20]. The half-life was 10 to 14 hours [21]. The majority of amantadine is excreted unchanged in urine. Amantadine accumulates in renal dysfunction [21]. In this study, we assessed renal function based on creatinine clearance and there was no decrement in renal function during and after study. In elderly or individual with renal dysfunction, it may cause CNS side effect such as delirium at high level of serum amantadine [19].

This study was designed to determine the short-term effect of IV amantadine on FOG. The study period was short and the washout period was not long. However, as stated in Table 3, the FOG of the AP group during the 2^nd^ admission was not better than that of the PA group, which means that the washout period did not affect the results of this study. The amantadine level at the time of the 2^nd^ admission was well below the therapeutic level, also.

And small sample size which the number of subjects was calculated with unilateral situation with low effect power was limitation in this study. This choice may explain the placebo effect of the drug, however based on our results that there is virtually no difference by amantadine infusion, required sample size should be very large. Considering our study enrolled very limited patients group who had severe (FOGQ score>10) and intractable (both ‘On’ and ‘Off’ state) FOG, we could not but limit the number.

This double blind, placebo-controlled study did not show the efficacy of IV amantadine compared to the placebo for FOG. The placebo effect may have obscured the benefit of IV amantadine in this short-term study. A longer duration study is needed to examine the possibility of delayed motor benefit.

## Supporting Information

Checklist S1 **CONSORT Checklist. Consort 20120 checklist of information to include when reporting a randomized trial.**
(DOC)

Protocol S1 **Trial Protocol. The Effect of IV amantadine on freezing of gait (FOG) resistant to dopaminergic therapy.**
(PDF)

Table S1 **Baseline characteristics of all patients (n = 8).**
(DOC)
