# Supplementary material for: Intravenous Amantadine for Freezing of Gait Resistant to Dopaminergic Therapy: A Randomized, Double-Blind, Placebo-Controlled, Cross-Over Clinical Trial
Source: PLoS One. 2012 Nov 19;7(11):e48890. doi: 10.1371/journal.pone.0048890 (PMC3501515; doi:10.1371/journal.pone.0048890)
Supplement: Protocol S1 — Trial Protocol. The Effect of IV amantadine on freezing of gait (FOG) resistant to dopaminergic therapy. (PDF) [file pone.0048890.s002.pdf]

ClinicalTrials.gov
Protocol Registration System

Send message to PRS

Protocol Registration Preview

Continue Select

The Effect of IV Amantadine on Freezing of Gait (FOG) Resistant to Dopaminergic Therapy

This study has been completed.

**Sponsor:**	Seoul National University Hospital
**Collaborators:**	
**Information provided by (Responsible Party):**	BS Jeon, Seoul National University Hospital
**ClinicalTrials.gov Identifier:**	NCT01313819

Purpose

Tp determine the effect of IV amantadine on dopaminergic-drug-resistant freezing of gait(FOG)in patients with Parkinson`s disease

Condition	Intervention	Phase
Parkinson`s Disease<br>Freezing of Gait	Drug: PK-Merz® 200mg/500ml inj(Amantadine) , Normal saline 500ml inj	Phase 4

Study Type: Interventional
Study Design: Treatment, Crossover Assignment, Double Blind (Subject, Caregiver, Investigator, Outcomes Assessor), Randomized, Efficacy Study

Official Title: A Double Blind, Placebo-controlled Study for the Effect of IV Amantadine on Freezing of Gait (FOG) Resistant to Dopaminergic Therapy

Further study details as provided by BS Jeon, Seoul National University Hospital:

Primary Outcome Measure:

- Change of FOGQ Score [Time Frame: 2 days for each drug] [Designated as safety issue: No]
  The relative change in the FOGQ scores were compared between the amantadine and placebo arms using the Wilcoxon signed rank test.

Secondary Outcome Measure:

- UPDRS III [Time Frame: 2 days for each drug] [Designated as safety issue: No]
  UPDRS III (motor) total score

- Side Effect [Time Frame: 2 days, 2 weeks after discharge] [Designated as safety issue: Yes]

- Patient Global Impression [Time Frame: 2 days, 2 weeks after discharge] [Designated as safety issue: No]
- 4*10m Walk Test [Time Frame: 2 days for each drug] [Designated as safety issue: No]

Enrollment: 10
Study Start Date: April 2011
Study Completion Date: December 2011
Primary Completion Date: October 2011

**Arms**	**Assigned Interventions**
Active Comparator: Group 1<br><br>Give IV amantadine first then IV placebo (normal saline) drug	Drug: PK-Merz® 200mg/500ml inj(Amantadine) , Normal saline 500ml inj<br><br>IV amantadine at 200 mg in 500 cm3 of saline solution or normal saline 500 cm3 given over a 3-h period, twice a day for 2 days along with the pre-existing dopaminergic and non-dopaminergic medication
Active Comparator: Group 2<br><br>Give IV placebo drug first then IV amantadine	Drug: PK-Merz® 200mg/500ml inj(Amantadine) , Normal saline 500ml inj<br><br>IV amantadine at 200 mg in 500 cm3 of saline solution or normal saline 500 cm3 given over a 3-h period, twice a day for 2 days along with the pre-existing dopaminergic and non-dopaminergic medication

1. Freezing of gait (FOG) is one of the most disabling symptoms of Parkinson`s disease.
   We experienced that severe FOG was markedly improved by IV amantadine in the patients who had Parkinson`s disease. But IV drug may have placebo effect. Therefore, We designed double blind, placebo controlled study to know whether IV amantadine is effective in the patient with dopaminergic-drug-resistant freezing of gait (FOG).
2. Cross over study design
   - Compare the change of FOGQ(freezing of gait questionaire) score from the baseline to IV amantadine and placebo drug
   - randomized assigned order of amantadine and placebo drug.
   - investigator of FOG: blinded to the order of drugs
   - each patient has IV drug for 2 days for each drug

Eligibility

Ages Eligible for Study: 30 Years to 80 Years
Genders Eligible for Study: Both

Inclusion Criteria

- age: 30-80 years
- idiopathic Parkinson's disease
- The patient must be taking optimised levodopa/DDI therapy (based on investigator's judgement) during OPD observation period, though the patient have FOG-Q score ≥ 10 points even though On-state.

Exclusion Criteria:

- "Off" freezing:The patient has improved FOG in "On" state
- clinically significant or unstable medical or surgical condition
- The patient has Parkinson plus like MSA, PSP, and PPFG, and secondary parkinsonism like NPH, vascular parkinsonism, postencephalitic parkinsonism, CO poisoning.
- history of seizure.

Contacts and Locations

Locations

Korea, Republic of

Seoul National University Hospital
Seoul, Korea, Republic of

Investigators

Principal Investigator:	Beom S Jeon, MD, PhD	Seoul National University Hospital

More Information

Publications:

Giladi N, Kao R, Fahn S. Freezing phenomenon in patients with parkinsonian syndromes. Mov Disord. 1997 May;12(3):302-5.

Giladi N. Medical treatment of freezing of gait. Mov Disord. 2008;23 Suppl 2:S482-8. Review. Erratum in: Mov Disord. 2008 Aug 15;23(11):1639-40.

Responsible Party: BS Jeon, Professor, Seoul National University Hospital
Study ID Numbers: H-1012-044-344
Health Authority: Korea: Institutional Review Board

Study Results

Participant Flow

Recruitment Details -- *Key information relevant to the recruitment process for the overall study, such as dates of the recruitment period and locations:*

From May 2011 to June 2011, all subjects were enrolled in outpatient clinic of Seoul National University hospital.patients who were diagnosed as Parkinson's disease, aged 30 to 80 years, with intractable FOG (FOG questionnaire score of ≥10 and FOG persisted on On and Off period, even though high doses of dopaminergic medication were first given)

Pre-Assignment Details -- *Significant events and approaches for the overall study following participant enrollment, but prior to group assignment:*

Although 12 patients assessed for eligibility, total 10 patients were randomized because 2 patients were excluded due to only off freezing and dementia.

Reporting Groups

	Description
Group1	Give IV amantadine first then IV placebo(normal saline) drug Intervention: Drug: PK-Merz® 200mg/500ml inj(Amantadine) , Normal saline 500ml inj
Group 2	Give IV placebo drug first then IV amantadine Intervention: Drug: PK-Merz® 200mg/500ml inj(Amantadine) , Normal saline 500ml inj

Overall Study

	Group1	Group 2
STARTED	5	5
COMPLETED	4	4
Not Completed	**1**	**1**
Protocol Violation	1	0
Adverse Event	0	1

Baseline Characteristics

Reporting Groups

	Description
Group1	Give IV amantadine first then IV placebo(normal saline) drug Intervention: Drug: PK-Merz® 200mg/500ml inj(Amantadine) , Normal saline 500ml inj
Group 2	Give IV placebo drug first then IV amantadine Intervention: Drug: PK-Merz® 200mg/500ml inj(Amantadine) , Normal saline 500ml inj

Baseline Measures

	Group1	Group 2	Total
Number of Participants	5	5	**10**
**Age Continuous** *[units: years]* *Mean ±Standard Deviation*	64.5 ± 8.9	66.3 ± 6.9	**65.4 ± 7.5**
**Gender, Male/Female** *[units: participants]*			
Female	3	2	**5**
Male	2	3	**5**
**Region of Enrollment** *[units: participants]* Korea, Republic of	5	5	**10**

Outcome Measures

1. Primary Outcome Measure:

**Measure Title**	**Change of FOGQ Score**
**Measure Description**	The relative change in the FOGQ scores were compared between the amantadine and placebo arms using the Wilcoxon signed rank test.
**Time Frame**	2 days for each drug
**Safety Issue?**	No

Population Description -- *Explanation of how the number of participants for analysis was determined. Includes whether analysis was per protocol, intention to treat, or another method. Also provides relevant details such as imputation technique, as appropriate:*

Total number of patients are 8 (Group 1 + Group 2). Amanatadine or placebo arm means clinical variables measured during amantadine or placebo injection, respectively. (because of Crossover design)

Reporting Groups

	Description
**Amantadine Arm**	**status (clinical variables) of all patients during injection of IV amantadine**
**Placebo Arm**	**status (clinical variables) of all patients during injection of IV normal saline (placebo)**

Measured Values

	Amantadine Arm	Placebo Arm
**Number of Participants Analyzed**	8	8
**Change of FOGQ Score** *[units: units on a scale]* Mean ± Standard Deviation	10.8 ± 3.2	9.1 ± 2.6

Statistical Analysis 1 for Change of FOGQ Score

**Groups**	Amantadine Arm, Placebo Arm
**Non-Inferiority/Equivalence Test**	Yes
**Method**	Sign test
**P-Value**	<0.05
**Mean Difference (Net)**	1.7

Additional details about the analysis, such as null hypothesis and power calculation:

The relative change in the FOGQ were compared between the amantadine and placebo arms using the Wilcoxon signed rank test

Additional details about a non-inferiority or equivalence analysis:

80% power.

Additional information, such as whether or not the p-value is adjusted for multiple comparisons and the a priori threshold for statistical significance:

[Not specified.]

Other relevant information, such as adjustments or degrees of freedom:

[Not specified.]

2. Secondary Outcome Measure:

**Measure Title**	**UPDRS III**
**Measure Description**	UPDRS III (motor) total score
**Time Frame**	2 days for each drug
**Safety Issue?**	No

Population Description -- *Explanation of how the number of participants for analysis was determined. Includes whether analysis was per protocol, intention to treat, or another method. Also provides relevant details such as imputation technique, as appropriate:*

Reporting Groups

	**Description**
**Amantadine Arm**	**status (clinical variables) of all patients during injection of IV amantadine**
**Placebo Arm**	**status (clinical variables) of all patients during injection of IV normal saline (placebo)**

Measured Values

	**Amantadine Arm**	**Placebo Arm**
**Number of Participants Analyzed**	8	8
**UPDRS III**<br>*[units: units on a scale]*<br>Mean ± Standard Deviation	19.5 ± 6.4	19.3 ± 6.6

Statistical Analysis 1 for UPDRS III

**Groups**	Amantadine Arm, Placebo Arm
**Non-Inferiority/Equivalence Test**	Yes
**Method**	Sign test
**P-Value**	<0.05
**Mean Difference (Net)**	0.2

Additional details about the analysis, such as null hypothesis and power calculation:

[Not specified.]

Additional details about a non-inferiority or equivalence analysis:

80% power

Additional information, such as whether or not the p-value is adjusted for multiple comparisons and the a priori threshold for statistical significance:

[Not specified.]

Other relevant information, such as adjustments or degrees of freedom:

[Not specified.]

3. Secondary Outcome Measure:

**Measure Title**	**Side Effect**
**Measure Description**	
**Time Frame**	2 days, 2 weeks after discharge
**Safety Issue?**	Yes

Population Description -- *Explanation of how the number of participants for analysis was determined. Includes whether analysis was per protocol, intention to treat, or another method. Also provides relevant details such as imputation technique, as appropriate:*

Reporting Groups

	Description
**Amantadine Arm**	**status (clinical variables) of all patients during injection of IV amantadine**
**Placebo Arm**	**status (clinical variables) of all patients during injection of IV normal saline (placebo)**

Measured Values

	Amantadine Arm	Placebo Arm
**Number of Participants Analyzed**	10	10
**Side Effect** *[units: participants]*	2	2

4. Secondary Outcome Measure:

**Measure Title**	**Patient Global Impression**
**Measure Description**	
**Time Frame**	2 days, 2 weeks after discharge
**Safety Issue?**	No

Population Description -- *Explanation of how the number of participants for analysis was determined. Includes whether analysis was per protocol, intention to treat, or another method. Also provides relevant details such as imputation technique, as appropriate:*

The number of patient who answered that Amantadine is better on FOG than placebo. - 3 subjects The number of patient who answered that placebo is better on FOG than amantadine. - 2 subjects

Reporting Groups

	Description
**Amantadine Arm**	**status (clinical variables) of all patients during injection of IV amantadine**
**Placebo Arm**	**status (clinical variables) of all patients during injection of IV normal saline (placebo)**

Measured Values

	Amantadine Arm	Placebo Arm
**Number of Participants Analyzed**	8	8
**Patient Global Impression** *[units: participants]*	3	2

5. Secondary Outcome Measure:

**Measure Title**	**4*10m Walk Test**
**Measure Description**	
**Time Frame**	2 days for each drug
**Safety Issue?**	No

Population Description -- *Explanation of how the number of participants for analysis was determined. Includes whether analysis was per protocol, intention to treat, or another method. Also provides relevant details such as imputation technique, as appropriate:*

Reporting Groups

	Description
**Amantadine Arm**	**status (clinical variables) of all patients during injection of IV amantadine**
**Placebo Arm**	**status (clinical variables) of all patients during injection of IV normal saline (placebo)**

Measured Values

	Amantadine Arm	Placebo Arm
**Number of Participants Analyzed**	8	8
**4*10m Walk Test** *[units: second]* Mean ± Standard Deviation	56.6 ± 17.7	99.8 ± 88.8

Reported Adverse Events

Reporting Groups

	Description
**Amantadine Arm**	status (clinical variables) of all patients during injection of IV amantadine
**Placebo Arm**	status (clinical variables) of all patients during injection of IV normal saline (placebo)

**Time Frame**	3 weeks
**Additional Description**	In the amantadine arm, 1 patient had transient hypertension and 1 patient had transient hypotension. In the placebo arm, 1 patient had transient delirium and hypertension who was withdrawn and 1 patient had transient hypertension. All subjects made a full recovery without residual complications. There was no worsening in renal function.

Serious Adverse Events

	Amantadine Arm	Placebo Arm
Total # participants affected/at risk	0/0	0/0

Other Adverse Events
Frequency Threshold Above Which Other Adverse Events are Reported: 5%

	Amantadine Arm	Placebo Arm
Total # participants affected/at risk	0/9 (0%)	0/9 (0%)

More Information

Certain Agreements:

Principal Investigators are NOT employed by the organization sponsoring the study.

There is NOT an agreement between the Principal Investigator and the Sponsor (or its agents) that restricts the PI's rights to discuss or publish trial results after the trial is completed.

Limitations and Caveats -- *Limitations of the study, such as early termination leading to small numbers of subjects analyzed and technical problems with measurement leading to unreliable or uninterpretable data:*

This study was designed to determine the short-term effect of IV amantadine on FOG. We cannot exclude the delayed benefit of amantadine on FOG.

Results Point of Contact:

Name/Official Title: BS Jeon
Organization: Seoul National University Hospital
Phone: 82-2-2072-2876
Email: brain@snu.ac.kr

Continue Select
