# Supplementary material for: Intravenous Amantadine for Freezing of Gait Resistant to Dopaminergic Therapy: A Randomized, Double-Blind, Placebo-Controlled, Cross-Over Clinical Trial
Source: PLoS One. 2012 Nov 19;7(11):e48890. doi: 10.1371/journal.pone.0048890 (PMC3501515; doi:10.1371/journal.pone.0048890)
Supplement: Table S1 — Baseline characteristics of all patients (n = 8). (DOC) [file pone.0048890.s003.doc]

**Table S1.** Baseline characteristics of all patients (n = 8)

| Variables | Baseline, mean ± SD (min-max) |
| --- | --- |
| Gender (male to female) | 4:4 |
| Age (y) | 65.4 ± 7.5 (53 - 75) |
| Age at onset of PD | 52.8 ± 6.7 (41 - 61) |
| Duration of PD (y) | 11.6 ± 3.79 (7 - 17) |
| Freezing of gait (FOG) duration (y) | 2.0 ± 1.6 (0.3 - 5) |
| Levodopa Equivalent Daily Dose (LEDD) (mg) | 1366.3 ± 282.3 (1050 - 1850) |
| Mini Mental Status Examination (MMSE) | 27.0 ± 1.4 (25 - 29) |
| Frontal Lobe Assessment Battery (FAB) | 15.0 ± 2.9 (10 - 18) |
| Initial UPDRS III “on” state | 23.3 ± 7.3 (16 - 40) |
| Initial FOGQ score | 16.9 ± 3.6 (13 - 21) |
